# Causes of Early Childhood Deaths in Urban Dhaka, Bangladesh

**DOI:** 10.1371/journal.pone.0008145

**Published:** 2009-12-03

**Authors:** Amal K. Halder, Emily S. Gurley, Aliya Naheed, Samir K. Saha, W. Abdullah Brooks, Shams El Arifeen, Hossain M. S. Sazzad, Eben Kenah, Stephen P. Luby

**Affiliations:** 1 Program on Infectious Diseases and Vaccine Science (PIDVS), International Center for Diarrhoeal Disease Research, Bangladesh (ICDDR,B), Dhaka, Bangladesh; 2 Department of Microbiology, Dhaka Shishu Hospital, Sher-e-Bangla Nagar, Dhaka, Bangladesh; 3 Departments of Biostatistics and Global Health, University of Washington School of Public Health and Community Medicine, Seattle, Washington, United States of America; 4 Departments of Biostatistics and Global Health, University of Washington School of Public Health and Community Medicine, Seattle, Washington, United States of America; BMSI-A*STAR, Singapore

## Abstract

Data on causes of early childhood death from low-income urban areas are limited. The nationally representative Bangladesh Demographic and Health Survey 2007 estimates 65 children died per 1,000 live births. We investigated rates and causes of under-five deaths in an urban community near two large pediatric hospitals in Dhaka, Bangladesh and evaluated the impact of different recall periods. We conducted a survey in 2006 for 6971 households and a follow up survey in 2007 among eligible remaining households or replacement households. The initial survey collected information for all children under five years old who died in the previous year; the follow up survey on child deaths in the preceding five years. We compared mortality rates based on 1-year recall to the 4 years preceding the most recent 1 year. The initial survey identified 58 deaths among children <5 years in the preceding year. The follow up survey identified a mean 53 deaths per year in the preceding five years (SD±7.3). Under-five mortality rate was 34 and neonatal mortality was 15 per thousand live births during 2006–2007. The leading cause of under-five death was respiratory infections (22%). The mortality rates among children under 4 years old for the two time periods (most recent 1-year recall and the 4 years preceding the most recent 1 year) were similar (36 versus 32). The child mortality in urban Dhaka was substantially lower than the national rate. Mortality rates were not affected by recall periods between 1 and 5 years.

## Introduction

Approximately 9.7 million children die annually from preventable causes [1]. Rapid urbanization has occurred throughout low income countries, where 80% of the world's largest cities are now located [2]. Moreover, a growing urban poor population in these countries is an increasing challenge for local health authorities [3]. Knowledge of the causes of child death is essential for appropriate health sector interventions [4]. In the South-Asian sub-continent, the decline in urban child mortality has been less than in rural areas [5]–[7]. The diversity and mobility of urban populations can make them harder to reach [5].

Verbal autopsy is an approach used to obtain cause of death by interviewing lay respondents on the signs and symptoms experienced by the deceased before death [8]. A nationally representative verbal autopsy study, the Bangladesh Demographic and Health Survey (BDHS) 1993–94 concluded that 24% of deaths among children under five years were associated with acute respiratory infections (ARI) and 19% were associated with diarrhea and/or possible diarrhea [9]. Using similar methodology but somewhat different case definitions the BDHS 2004 concluded that possible serious infection (31%) and ARI (21%) were responsible for most of the deaths [10]. BDHS 2007 reported that 63 under five children died per 1,000 live births in urban areas compared to 77 deaths in rural in the ten year period preceding the survey [7]. This pattern was similar to BDHS 2004 [10]. Verbal autopsy studies are common and although they often use different recall durations, few data are available to assess the validity of different recall durations.

Dhaka *Shishu* (Children's) Hospital and *Shishu Shaystha* (Child Health) Foundation Hospital are two leading pediatric hospitals in Bangladesh located near each other in the central and north-west sectors of Dhaka city. This analysis is part of a broader study among children under-five years who were admitted to these two hospitals with a diagnosis of pneumonia, meningitis or sepsis, and the disease burden in the children under age five years who live in the contiguous overlapping neighborhoods who most commonly use these two hospitals. The objectives of this analysis were to estimate the rates and causes of death among children under age 5 years in the catchment population of these hospitals in urban Dhaka, Bangladesh, and to compare the under-4 child mortality rates derived from two recall periods, one year preceding the interview versus the four years preceding the recent one year.

## Methods

### Study Population

We conducted an initial community health and demographic survey from May to August 2006 and a follow up survey within the same households and in the same months in 2007. Some different results from the initial survey were published previously [11].

We enrolled 70 clusters, each consisted of 100 households. Clusters represented neighborhoods that commonly used Dhaka *Shishu* (child) and *Shishu Shaystha* (Child Health) Foundation Hospitals. To select a representative sample of the population served by these hospitals, we allocated the number of clusters to each hospital proportional to the number of children admitted per year to those two hospitals. For selecting each cluster, field workers listed the 20 most recent children under the age of 5 years admitted to the hospital who had a provisional diagnosis of respiratory illness, pneumonia, meningitis or sepsis. The field workers selected a patient using a random number table and asked if the patient lived within 60 minutes travel time to the hospital by any means of transportation as described by the patient's guardian. If they lived within 60 minutes, they were selected as a cluster, if they did not then another random number and another child was selected. We chose a 60 minutes traveling time as an inclusion criterion because based on preliminary discussions with patients at the two hospitals, most lived within 60 minute traveling time.

For each selected child, the field workers located the patient's home and marked it as the starting point for a cluster. The field team noted the patient's household as the ‘index child’, then skipped the household of the index child and next closest five households. Fieldworkers skipped neighbors of index cases to avoid enrolling extended family members or persons who may have been involved in a disease outbreak. After skipping these households, the first eligible household was the closest one with at least one child under the age of five years and/or any child under five years of age who died in the one year prior to the survey date. The field workers sought informed consent for enrolment in the study and then administered a structured questionnaire. Field workers traveled to the next closest household without any further skipping of households under the same inclusion criteria of at least one child under the age of five years and/or any child under five years of age who died in the one year prior to the survey date. Traveling to next closest household continued till 100 households identified. From May to August 2006, study workers identified seventy (70) ‘index children’ for community starting points and sought one hundred (100) households from each of these starting points for a targeted sample size of 7000 households. We chose 70 index children to represent a reasonable diversity of neighborhoods that used the hospitals. Field workers visited sampled households repeatedly including evenings and weekends and made appointments, if necessary, to return at a time that was convenient to study subjects to maximize participation.

After one year, the study workers once again visited the same households if the survey inclusion criteria were still fulfilled. Because the child mortality rate, based on analysis of the one year recall in the 2006 survey was low, for the 2007 survey we inquired about deaths among children under age 5 years during the five (5) years prior to the survey date during the follow-up survey, to be able to compare the rates to other reports [6] using a similar recall period. During the follow up survey, the study workers replaced all households that had either out migrated or whose children had matriculated out of the <5 year age group with the closest available household with children in the age group that satisfied the inclusion criteria.

### Instruments and data collection

Trained field workers initially administered a questionnaire including demographic information of all household members, materials of household infrastructure, education, occupation, ownership of household assets, household utilities, and access to water, sanitation and hygiene facilities.

In households where a child less than 5 years of age had died, the field workers administered separate structured verbal autopsy questionnaires for either neonatal (≤28 days) or child (29 days to <5 years) deaths. Only female field workers interviewed mothers about neonatal deaths because of gender-sensitive questions surrounding childbirth. The field workers interviewed either the mother of the deceased or the next closest available family member who was knowledgeable about the events leading up to the death. We followed BDHS verbal autopsy methodologies that have been used previously in Bangladesh and other countries to collect information on causes of death [10], [12]–[13]. We estimated that the verbal autopsy questionnaire took average 90 minutes per interview.

### Diagnosis of causes of death

Diagnoses of causes of deaths were coded according to the International Statistical Classification of Diseases (ICD-10) codes [14]. Separately and independently, two physicians trained in verbal autopsy coding, initially assigned two codes of causes of death for each deceased child questionnaire, the immediate cause of death and the underlying cause of death as per the diagnosis criteria (table 1). When the study coordinator found that the two physicians initially disagreed on a child's diagnosis, then the physicians discussed the difference and reached consensus on the most appropriate code. To assign the unique cause of death from the immediate and underlying causes, the physicians prioritized the main cause of diseases. For example, when the physicians found that pneumonia, sepsis or meningitis was either immediate or underlying cause, they considered it as the unique cause. This approach replicates the approach used by the Bangladesh Demographic and Health Survey to permit comparison of the cause specific mortalities [4], [10]. We compared all cause specific neonatal and under-five mortality proportions specifically to the national Bangladesh Demographic and Health Survey 2004 data, the most recent year that included verbal autopsy.

**Table 1:** Criteria for assigning causes of death, Dhaka city, Bangladesh, 2006 & 2007.

Diagnosis	Criteria	
	Neonate (0-28days)	Child (29 days-5 years)
Congenital disease or anomaly	Any congenital abnormality leading to death e.g. congenital mega colon	Any congenital abnormality leading to death For example- Congenital heart disease Congenital malformation of brain Claft palet Sacrococcigeal teratoma
Accident	Any accident that led to death	Any accident led to death, for example drowning
Preterm birth/low birth weight	Gestational age <37 completed weeks, and/or weight <2.5 kilogram	N/A
Delivery complications	At least one criteria matched- Antepartum hemorrhage, Toxemia of pregnancy Premature rupture membrane Prolonged labor Obstructed labor Cephalopelvic disproportion Malpresentation	N/A
Diarrhoea	Passage of loose watery stool more than 3 times per day that led to death	Passage of loose watery stool more than 3 times per day that led to death
Respiratory diseases	Fast breathing and chest indrawing as key symptom and associated with one or more of the following-Difficulty breathing, Stridor Cyanosis Cough Fever	Fast breathing and/or chest indrawing as key symptom and associated with one of more of the following-Difficulty breathing, Stridor Cyanosis Cough Fever
Sepsis	Key symptoms were-Any fever Hypothermia Lethargy Reluctant to feed Associated with any of the following symptoms-Convulsion Fast breathing Bleeding Abdominal distention	Key symptoms were-Any fever Hypothermia LethargyReluctant to feed Associated with any of the following symptoms-Convulsion Fast breathing Bleeding Abdominal distention
Meningitis	Key symptoms-Fever and convulsion Associated with any of the following symptoms-Bulge fontanel Reluctant to feed Drowsiness	Key symptoms-Fever and convulsion Associated with any of the following symptoms-Neck rigidity Bulge fontanel Reluctant to feed Drowsiness
Birth asphyxia	Any of the following symptoms that let to death- Delayed crying after birth Not able to breath after birth Respiratory distress since birth	N/A
Protein energy malnutrition	N/A	Any of the following symptoms that let to death- Very thin Pallor History of weight loss Monkey face Swollen leg
Other	Any of the following- Birth injury Aspiration of milk Neonatal jaundice	Any of the following- Acute renal failure Acute glomarulonephritis Nephrotic syndrome Malignancy Enteric fever, diabetes Cerebral palsy Cirrhosis
Not identified	Inconsistent and incomplete history	Inconsistent and incomplete history

### Data management

The trained physicians reviewed the verbal autopsy questionnaires within 24 hours of data collection. Field researchers returned to households to collect missing or additional important information if required. Supervisor physicians directly observed the interviewing techniques of 5% households for quality control on interview technique.

### Data analysis

Because the household enrollment criteria were different for this study then for standard demographic and health surveys, the analysis of these data was also somewhat different. Mortality estimates were based on Kaplan-Meier estimates of the survival probability by age. Confidence intervals were based on the asymptotic variance of ln(-ln(S(t)), where S(t) denotes the probability of surviving to age t. The use of survival analysis facilitates comparison of our results to those of the Bangladesh Demographic and Health Survey, which used life-table methods to calculate survival probabilities. All survival analysis was performed in Intercooled Stata version 9.2 (© StataCorp LP), and all methods used are described elsewhere [15].

Neonatal and under-5 mortality from the 2006 and 2007 surveys with one-year recall periods were calculated from the Kaplan-Meier estimate of the survival probability at ages 28 and 1826 days, respectively. The estimated risk of death at each age was multiplied by 1,000 to estimate mortality per thousand live births.

We compared age and cause specific death rates among children reported to have died in the year preceding the interview in 2007 to children reported to have died >1 and <5 years preceding the 2007 interview using Kaplan-Meier estimates of the survival probability. The survey collected birth information for all children under age five at the time of the survey and death information from all children who died by age five within the five years of the survey date. To ensure that inclusion criteria for living and deceased children were the same, deceased children born more than five years prior to the survey date were excluded from the analysis. Since deaths during the recall period >1 to ≤5 years prior to the survey date only captures information on children up to age four we calculated overall and cause-specific neonatal, child (1–48 months), and under-4 mortality rates separately using recall data from ≤1 year prior to the survey date and from >1 to ≤5 years prior to the survey date (4 years duration). For the ≤1 year recall period, children entered the risk pool one year before the survey date or on their date of birth, whichever was later. For the >1 to ≤5 year recall period, children entered the risk pool on their date of birth and left the risk pool on their date of death or one year prior to the survey date, whichever was earlier. The Kaplan-Meier estimates of the risk of death by 28 days and 1461 days were multiplied by 1000 to estimate neonatal and under-4 mortality, respectively, per thousand live births. The risk of death between the ages of 1 and 48 months was based on the Kaplan-Meier estimate of the risk of death by age 1461 days among children who survived to age 29 days. Cause-specific mortality was calculated by repeating this analysis with deaths due to each cause counted as failure events and deaths from all other causes counted as censored events. To compare the overall survival estimates from the two recall periods, we tested the null hypothesis of equal survival functions with a log-rank test.

### Ethics

Adult respondents in participating households and caregivers of living and deceased children provided informed written consent. The study protocol was reviewed and approved by the Ethical Review Committee of the International Centre for Diarrheal Disease Research, Bangladesh (ICDDR,B).

## Results

During the initial survey in 2006, from the 7000 identified households that met the enrolment criteria, field workers completed interviews in 6971 (99.6%). Conducting the follow up survey in 2007, we revisited all 6971 households but had to replace 51% (3521) of households. We replaced 492 households because their children were now all older than the five year age limit and 3029 households because residents had moved. We included an additional 49 households that were still within the cluster boundary and had a child under the age of 5 years during survey date; die in last 5 years even though they did not have a child under age 5 years living at the household at the time of the survey.

The initial survey in 2006 identified 58 deaths (20 [34%] neonatal and 38 child) among children under age 5 years in the one year prior to the survey date, compared with 61 deaths (32 [53%] neonatal and 29 child) in the 2007 survey. The five year recall period prior to survey date during the follow up survey identified 267 under-five deaths (mean 53 deaths per year, SD±7.3) in which 56% were neonatal deaths.

In the one year prior to the two surveys the neonatal mortality rate was 15.1 (95% CI: 11.5, 19.9) and under-five mortality rate was 34.0 (95% CI: 28.4, 40.8) per 1000 live births.

**Figure 1:**
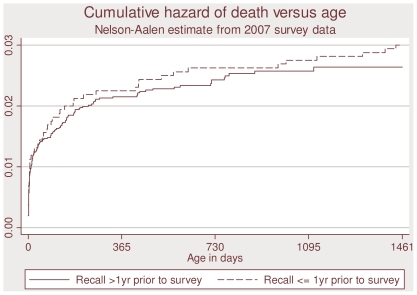
Comparison of mortality in different recall periods, urban Dhaka, Bangladesh.

The overall under-4 mortality rates per thousand live births calculated for 1 year recall duration similar to those based on the duration >1 to ≤5 years (36 versus 32, p = 0.53) (table 2). Neonatal and child (1–48 months) mortality rates in the two recall periods were also similar (20 and 18 for neonatal, p = .62, and 17 and 14 for child, p = .71) (table 2). This similarity is clearly visible in the cumulative hazard curves in figure 1.

**Table 2:** Disease specific mortality, urban Dhaka, Bangladesh.

Disease		Preceding 1 year, 2007			Deaths in preceding >1 to ≤5 years	
	Under-4 deaths*Rate (CI)Number of deaths	Neonatal deaths* (0–28 days)Rate (CI)Number of deaths	Child deaths** (1–48 months)Rate, (CI)Number of deaths	Under-4 deaths*Rate (CI)Number of deaths	Neonatal deaths* (0–28 days)Rate (CI)Number of deaths	Child deaths** (1–48 months)Rate (CI)Number of deaths
Respiratory disease	8.1 (4.7, 13.9)13	2.5 (0.9, 6.6)4	5.6 (2.9, 10.7)9	9.8 (7.5, 12.8)57	4.2, (2.9, 6.1)27	5.7, (3.9, 8.2)30
Sepsis	13.0 (8.5, 19.9)21	6.2 (3.4, 11.6)10	6.8 (3.8, 12.3)11	5.9 (4.2, 8.3)35	3.4 (2.3, 5.2)22	2.5 (1.4, 4.4)13
Others	15.5 (10.5, 22.8)25	11.2 (7.1, 17.7)18	4.4 (2.1, 9.1)7	16.1 (13.0, 19.9)94	10.4 (8.2, 13.2)68	5.7 (3.7, 8.8)26
Total	36.2 (28.1, 46.4)59	19.8 (14.0, 27.9)32	16.7 (11.5, 24.2)27	31.6 (27.2, 36.6)186	17.9 (15.0, 21.5)117	13.9 (10.8, 17.8)69

Note: *Deaths per thousand live births (for neonatal and under-4 mortality); **deaths per thousand children who survive to 29 days (for child deaths).

Sepsis (13 deaths per 1000 live births, 36%) and respiratory disease (8 deaths per 1000 live births, 22%) were the leading causes of under-4 mortality in the one year preceding the survey. Similarly sepsis (6 deaths per 1000 live births, 19%) and respiratory disease (10 deaths per 1000 live births, 31%) were leading causes of death recalled >1 year and ≤5 years previously. Comparing cause-specific mortality in the two durations, the only statistically significant difference found was higher mortality from sepsis in the ≤1 year recall period (p = 0.01). However, this difference was borderline significance after correction for multiple comparisons. There was no other statistically significant difference in overall, respiratory, or other-causes neonatal, child, or under-4 mortality (all p-values >0.3) (table 2).

For neonatal deaths, over one-third of the urban cause of deaths was birth asphyxia followed by another one-third of possible serious infections which was broadly similar to results from the Bangladesh Demographic and Health Survey in 2004 (Table 3). Among children under-five in this community in urban Dhaka, we estimated that over 30% of deaths were caused by possible serious infections followed by ARI which is again similar to Bangladesh Demographic and Health Survey 2004 findings (Table 3).

**Table 3:** Comparison of causes of under-five mortalities in urban Dhaka 2006–2007 with BDHS 2004.

Causes of death	Death Surveillance of Urban Dhaka, 2006–2007 (%)	BDHS 2004 (%)
**Neonatal deaths (0–28 days)**		
Neonatal tetanus	0.0	4.2
Congenital abnormality	2.0	5.1
Early neonatal or pregnancy/delivery related^a^	0.0	0.0
Early prenatal	0.0	0.0
Birth asphyxia	36.0	21.1
Birth injury	12.0	3.9
Diarrhea	0.0	1.2
ARI	10.0	10.3
ARI and diarrhea	0.0	0.5
Possible serious infection^c^	32.0	33.2
Premature birth/LBW	6.0	10.9
Malnutrition	0.0	0.0
Other causes^d^	0.0	2.3
Unspecified	2.0	3.9
Undetermined	0.0	3.4
**Total deaths (n)**	**50**	**326**
**Under-five deaths**		
Neonatal tetanus		2.3
Congenital abnormality	0.9	2.8
Early neonatal or pregnancy/delivery related^a^		
Early prenatal		
Injury	2.6	4.1
Drowning	2.6	3.0
Others		1.1
Birth asphyxia	15.5	11.7
Birth injury	5.2	2.2
Measles	0.9	0.3
Measles followed by ARI or diarrhea		0.3
Diarrhea	6.9	5.1
ARI	22.4	21.1
ARI and diarrhea	5.2	1.8
Possible serious infection^c^	30.2	31.2
Premature birth/LBW	2.6	6.5
Malnutrition	1.7	3.6
Other causes^d^	1.7	1.6
Unspecified	3.4	3.2
Undetermined	0.9	2.3
**Total deaths (n)**	**116**	**587**

a Includes all deaths in the first 3 d of life and deaths after 3 d of life that were considered due to congenital anomaly, pre-maturity and complications of delivery.

b Confirmed diagnosis and Possible diagnosis merged together.

c Possible serious infections include possible ARI and diarrhea.

d Causes includes umbilical hemorrhage (1), hemorrhage disorder of newborn (3), otitis media (1), neonatal malnutrition (1), intestinal obstruction (1), aspiration pneumonia (1), and congenital heart disease (1).

## Discussion

Neonatal mortality 15 deaths per 1000 live births and under five mortality 34 deaths per 1000 live births estimated were much lower in this study population in urban Dhaka compared to national estimates [6]–[7]. In addition the Government of Bangladesh commissioned an Urban Health Survey in 2006 which selected a representative sample of slum and non-slum communities in the six largest cities in Bangladesh. The rates of neonatal and under five mortality in our study community were again much lower compared to national urban data (2006) in which the district municipalities neonatal mortality rate was 43 and under five mortality rate was 61 per 1000 live births. Child mortality rates in this study community were similar to the national non-slum urban data (neonate 31 and under-five 31 deaths per thousand live births) but lower than the slum data (neonate 44 and under-five 81 per thousand) [16]. Although we did not capture the information to explicitly classify communities, we estimate that approximately 30% of survey households were in slums. Therefore, we would expect a mortality rate between the national slum and non-slum rates, but instead we measured a rate closer to national non-slum urban data. A possible reason for a lower than expected mortality could be that either of the two large pediatric hospitals are within one hour traveling distance and therefore, available to children who have a life threatening but treatable illness. Other possible reasons include, the availability of better outpatient services from a range of providers in an urban environment, migration out after experiencing the death of a child, and higher levels of education [11] that may facilitate a healthier environment and more effective decisions when a child is ill.

Leading causes of under-five death in this community included respiratory disease and sepsis (table 2). Over the last several years mortality from diarrhea has declined but ARI has remained steady in Bangladesh and in other settings [4], [6], [9]–[10], [12], [17]–[18]. Reducing mortality from respiratory illness and neonatal mortality will be important to achieve the millennium development goal for child survival [19].

Estimated rates of under-four mortality were similar for recall periods of 1 year and >1 to <5 years. This suggests that child deaths are sufficiently memorable for caretakers and/or mothers to recall up to five years as followed by Demographic and Health Surveys (DHS) and other mortality studies [4], [6], [10]. Indeed, it would be worth evaluating recall periods over five years because longer recall durations can increase the efficiency of data collection and statistical power of the results.

The study had limitations. Verbal autopsy data are limited to the symptoms that family members can recall. They do not include a systematic medical assessment of the ill child, physical exam, laboratory records or postmortem evaluation. Although some causes of death are likely well captured by verbal autopsy for example drowning or severe diarrhea, other causes with less specific presentations including immune deficiencies and congenital metabolic problems are less likely to be recognized. Indeed, with the small sample of deaths in this study, there was limited statistical power to identify less common causes of death.

A second limitation is that a large proportion of the population migrated out of the study area. If households that experienced a child death, were more likely to migrate out of the community, then we may have under-estimated child mortality in these communities. However, most people who migrate in these communities migrate to nearby neighborhoods, often still within the hospitals catchment, and the socio-economic status of households that migrated in were similar to the households that they replaced in the study, so we believe they are a reasonable proxy.

A third limitation is that the enrollment criteria for households would have excluded some households which had a child death more than one year ago, and so underestimated mortality. In the 2006 survey, houses were selected if they had children under five or a child under five who died within one year of the survey date. In the 2007 survey, these same households were visited and asked about child deaths during the longer recall period. Households with a child who died under age five more than one year ago would only be included if they had a living child or a child who died within one year of the 2006 survey. Thus, some deaths from the extended recall period would be excluded from the survey because their household did not meet the inclusion criteria. However, this limitation only affects the estimates of mortality from recall periods >1 year. Moreover, the small differences in cumulative hazards between the ≤1 year recall period and the >1 year recall period suggests that impact of this limitation on the mortality estimates was minor.

A fourth limitation is that deaths among children under five within five years of the survey were included, whereas living children were included only if they were under five at the time of the survey. This creates selection bias in the direction of overestimating mortality because deceased children were included who would not have been included had they survived. We addressed this problem in the analysis by excluding children born more than five years before the survey date. In effect, our estimates of mortality are for an open cohort of children born in the last five years in the study area. Any household with one of these children, living or deceased, would have been eligible for the survey, and we would have data on all such children from any household that participated in the study. The mortality estimates from the <1-year recall period are similar to those that would be obtained from a synthetic cohort, whereas the mortality estimates from the >1-year recall period are those of an actual open cohort. The two methods produce similar mortality estimates when age-specific mortality rates are stable over time. Since the mortality estimates from the two periods are indeed similar (Figure 1), comparison with the BDHS estimates is reasonable despite the methodological differences.

A fifth limitation is that this study population represents a single geographic area. It is neither representative of all of Dhaka, nor of urban Bangladesh generally. Because it is only one site, limited inferences can be drawn regarding the relationship between available characteristics and child survival. However, the study population represents a large socio-economically diverse population and suggests that access to appropriate clinical care may contribute to improved child survival.

Although these data suggest that under-five child survival is better in this study community in urban Dhaka than in the country as a whole, however the mortality is still high. Improving child survival in Bangladesh will require reducing serious child respiratory disease, reducing neonatal deaths, and extending effective health services to both rural and urban areas.
